# Leber’s Congenital Amaurosis: Current Concepts of Genotype-Phenotype Correlations

**DOI:** 10.3390/genes12081261

**Published:** 2021-08-19

**Authors:** Chu-Hsuan Huang, Chung-May Yang, Chang-Hao Yang, Yu-Chih Hou, Ta-Ching Chen

**Affiliations:** 1 Department of Ophthalmology, Cathay General Hospital, Taipei 106, Taiwan; charleswain.h@gmail.com (C.-H.H.); ychou51@ntu.edu.tw (Y.-C.H.); 2 Department of Ophthalmology, National Taiwan University Hospital, Taipei 100, Taiwan; chungmay@ntu.edu.tw (C.-M.Y.); chyangoph@ntu.edu.tw (C.-H.Y.); 3 Department of Ophthalmology, College of Medicine, National Taiwan University, Taipei 100, Taiwan

**Keywords:** Leber’s congenital amaurosis, genotype-phenotype correlations, GUCY2D, RPE65, CRB1, CEP290, RDH12

## Abstract

Leber’s congenital amaurosis (LCA), one of the most severe inherited retinal dystrophies, is typically associated with extremely early onset of visual loss, nystagmus, and amaurotic pupils, and is responsible for 20% of childhood blindness. With advances in molecular diagnostic technology, the knowledge about the genetic background of LCA has expanded widely, while disease-causing variants have been identified in 38 genes. Different pathogenetic mechanisms have been found among these varieties of genetic mutations, all of which result in the dysfunction or absence of their encoded proteins participating in the visual cycle. Hence, the clinical phenotypes also exhibit extensive heterogenicity, including the course of visual impairment, involvement of the macular area, alteration in retinal structure, and residual function of the diseased photoreceptor. By reviewing the clinical course, fundoscopic images, optical coherent tomography examination, and electroretinogram, genotype-phenotype correlations could be established for common genetic mutations in LCA, which would benefit the timing of the diagnosis and thus promote early intervention. Gene therapy is promising in the management of LCA, while several clinical trials are ongoing and preliminary success has been announced, focusing on RPE65 and other common disease-causing genes. This review provides an update on the genetics, clinical examination findings, and genotype-phenotype correlations in the most well-established causative genetic mutations of LCA.

## 1. Introduction

Inherited retinal dystrophies (IRDs) are a group of diseases caused by genetic mutations that are characteristic of photoceptor dysfunction and eventual death of retinal cells. Different extents of retinal degeneration result in widely varied presentations, from milder night blindness or color blindness to profound visual impairment [1]. Among the diverse phenotypes and genotypes within IRDs, Leber’s congenital amaurosis (LCA) is one of the earliest and most severe forms of IRDs. In 1869, Dr. Theodore Leber first described severe visual impairment in infants with nystagmus and poor pupillary light reflex, which were recognized as typical presentations of the later-named LCA [2]. LCA represents 5% of all IRDs and has a prevalence of 1/81,000 to 1/30,000. It also accounts for 20% of blindness in school-aged children [3,4,5,6].

To establish the diagnosis of LCA in children with suspected IRDs, detailed ophthalmic history, imaging studies, electrophysical examinations, and, most importantly, molecular confirmation is needed. Compared to other retinal dystrophies, LCA is even more heterogeneous in its disease course and presentation. A milder phenotype of LCA with relative visual preservation has been considered as early-onset severe retinal dystrophy, although their genetic basis overlaps significantly [7]. Owing to the diversity in genetic background, disease presentation, and early onset in infancy, it is challenging but important for ophthalmologists to obtain a definitive diagnosis in preliminary consultations. We searched the PubMed database with the keywords “Leber’s congenital amaurosis” and each specific gene (i.e., *GUCY2D*) for studies published before 30 June. We included articles published in Science Citation Index (SCI) journals and focused on reporting the clinical presentation of LCA cases with specific genetic mutations. Thus, this review aims to summarize the clinical manifestations and genetic updates of LCA, as well as show the genotype-phenotype correlations in LCA to promote early diagnosis and facilitate bridging therapies in the near future. 

## 2. Clinical Manifestation 

Typically, patients with LCA present with poor vision and nystagmus or the absence of fixation at as early as 6 weeks of age. Manifestation of vision loss in patients with LCA ranges from functional visual acuity to light perception only, and approximately three-quarters of these are stationary. Transient fluctuation in vision with delayed visual maturation is occasionally observed in the first or second decade of life until progressive decline occurs [3]. The most commonly presented refractive error is hyperopia, especially with the *GUCY2D* mutation. The patient may present with photophobia or nyctalopia, which could be gene specific, as described by Hanein et al. [8]. Similar to the presentation of other diseases resulting in congenital blindness, oculodigital signs are common in patients with LCA. Keratoconus and juvenile cataracts are other associated ocular features. Mental retardation could be concurrent in about one-fifth of syndromic or non-syndromic LCA cases. Renal and olfactory dysfunction may be present and are associated with mutations in specific genes [9].

Electrophysical studies are important in assessing patients with suspected LCA in their early life. Although a non-detectable electroretinogram (ERG) response is typical in LCA, a residual cone response could be detected with mutation in *GUCY2D*, and a residual rod response in *RPE65* [10]. Fundoscopic imaging provides diagnostic clues, including peripheral pigmentary retinopathy, central maculopathy with or without bull’s eye pattern, or even frank macular atrophy. Depigmented fundus, reduced autofluorescence, whitish RPE spot, vascular attenuation, pseudopapillary edema, or coats-like vasculopathy have also been reported in the fundus of LCA cases [11]. Originally, time-domain optical coherence tomography (OCT) was rarely performed because of the commonly presenting nystagmus and young age, but the improvement in spectral-domain OCT technology has enhanced its application in this patient group [12]. The retinal structure revealed in OCT is dependent on age and may show specific abnormalities with a certain genotype (*CRB1*, *GUCY2D*). The disrupted anatomical structure of the retina includes decreased thickness in different layers, especially in the outer nuclear layer (ONL), loss of integrity in the ellipsoid zone, and disorganized macular atrophy [13]. The central foveal structure is relatively preserved in specific genetic mutations, such as *GUCY2D* and *RPE65*, which indicates a possible therapeutic window for genetic remodeling [14]. 

In summary, typical findings defining LCA includes (1) early and severe reduction of vision (from non-light perception to 20/400) associated with nystagmus in a large number of patients; (2) fundus appearance, ranging from normal, maculopathy, to typical retinitis pigmentosa (RP)-like abnormalities; and (3) severely reduced to non-detectable full field ERG responses [9]. Because of the clinically overlapping presentations, the differential diagnosis of LCA includes both syndromic and non-syndromic diseases. ERG is helpful in distinguishing it from achromatopsia, which has a normal rod response, and congenital stationary night blindness, which preserves the a-wave. The pupillary light reflex could be absent when combined with optic nerve hypoplasia. Specific systemic associations could be found in Batten disease, albinism, Joubert syndrome, peroxisome disease, Alstrom disease, and cobalamin C deficiency [15,16]. 

## 3. Genetic Features 

Linkage analysis, identity-by-descent mapping, and the candidate gene approach are used to identify the mutation loci in LCA cases [9]. Since the recognition of the first gene, *GUCY2D*, in LCA cases, the Online Mendelian Inheritance (OMIM) database has identified 19 types of LCA with mutations in specific genes [17]. The incorporation of more efficient next-generation sequencing techniques, such as whole exome sequencing and whole genome sequencing, has enabled the exploration of more causative genes. So far, there have been 19 other genes considered to be responsible for the LCA phenotype according to the OMIM database (Table 1).

With the advances in molecular genetics, approximately three-quarters of the LCA cases could be identified with specific mutations [10,18]. Most of the causative mutations are inherited by an autosomal recessive pattern, with the exception of *CRX*, *IMPDH1*, and *OTX2* [10]. These widely ranged mutations are involved in different aspects of maintaining normal retinal function or health. The common disease-causing mechanisms are as follows: phototransduction (*GUCY2D*, *AIPL1*, *RD3*, *KCNJ13*), retinoid cycle (*RPE65*, *LRAT*, *RDH12*), ciliary transportation (*LCA5*, *CEP290*, *RPGRIP1*, *SPATA7*, *TULP1*, *IQCB1*), photoreceptor morphogenesis (*CRX*, *CRB1*, *GDF6*, *PRPH2*), guanine synthesis (*IMPDH1*), and photoreceptor differentiation (*OTX2*) [9,10]. The mechanism involved in the LCA phenotype of recently identified mutations in *USP45* (LCA type 19) and other genes is still controversial and further studies are required [19]. 

Common mutations, usually occurring with a frequency of approximately 10% or higher, include *GUCY2D*, *RPE65*, *CRB1*, *CEP290*, and *RDH12* [9,20]. The frequency could vary according to ethnical background of the patients. Mutations in *CEP290*, *GUCY2D*, and *RPE65* are generally more frequent in Caucasian populations [20,21,22]. On the other hand, *CRB1*, which accounts for 13.6% of LCA cases, has been reported as the leading causative genetic mutation in Chinese cohorts, followed by *GUCY2D* [23]. Meanwhile, there was a significantly less common mutation in *CEP290* than in the European cohort, but more in *RPGRIP1*. The most common variant of the *CEP290* mutation found in the European population, c.2991+1655A>G, was not detected in the Chinese cohort [23]. *CRB1*, *NMNAT1*, and *RPGRIP1* were reported as the most common mutations in a Japanese cohort [24]. *CEP290* and *GUCY2D* were the leading causative mutations in Australia, followed by *NMNAT1* [25]. Moreover, these known mutations common in European and East Asian populations only accounted for 24% of LCA cases in the Saudi Arabian population, and several novel mutations have been discovered in this population [26]. These findings revealed regional differences in the genetic backgrounds of LCA cases. 

## 4. Genotype-Phenotype Correlation

Although patients with LCA exhibit an extended range of intra- and interfamilial heterogenicity, genotype-phenotype correlations have been identified in several common mutations [15,16]. Establishing these correlations could facilitate the confirmation of molecular diagnosis by narrowing the range of responsible LCA genes when a specific phenotype is found. The incorporation of multimodal images, including fundus photography, autofluorescence, OCT, and ERG, may reveal more detailed information and improve the predictability of specific molecular defects (Table 1). 

### 4.1. GUCY2D

*GUCY2D*, the first gene that was identified to be correlated with LCA, is localized on chromosome 17p13.1, and encodes retinal guanylate cyclase-1, which is involved in photoreceptor recovery phase in phototransduction [27]. *GUCY2D* is expressed in the photoreceptor outer segment with a higher level in cone cells than in rod cells [28]. The *GUCY2D* mutation accounts for 6–21% of LCA cases. *GUCY2D*-associated LCA cases are known to have very poor but stationary vision early in life, with nystagmus, oculodigital signs, and prominent photophobia [8,29]. Approximately 50% of cases present with vision worse than counting fingers, while only one-quarter of cases have vision measurable by the Snellen Chart [30]. While nonsense and some missense mutations in *GUCY2D* result in complete deficiency of enzyme function [9], Bouzia et al. found that a group of cases with a *GUCY2D* mutation on exon 2, which encodes the extracellular domain, had a milder phenotype with better visual acuity [31]. Although nyctalopia is less frequently reported, Bouzia et al. reported an incidence of 38% in their case series [31]. High hyperopia above +5 D, cataracts, and keratoconus are common ocular features [8,31]. Fundus image and autofluorescence appear nearly normal in most cases in childhood, but Jacobson et al. recently found that central retinal pigment epithelium (RPE) change and peripheral pigmentation could be noted as early as the third decade of life, which was much earlier than that in previous reports [32]. Despite severe visual impairment in these cases, a relatively preserved retinal structure with normal ONL thickness is observed on OCT [14]. The fovea bulge, representing the cone outer segment, is less prominent, and the ellipsoid zone (EZ) is intact, but with reduced intensity [30]. The ONL thickness in the fovea has been shown to decrease at a rate of 0.90 µm per year, while it remained stable in the rod-rich superior retina region [32]. In patients presenting with a central RPE change in fundus autofluorescence, the corresponding EZ loss could be found on OCT [31]. The cone response in ERG is generally diminished, while approximately 25% of cases have a preserved but attenuated rod response. Previous studies have demonstrated a detectable rod response on the full-field stimulation test as well [13,33]. This functional finding corresponded with the relatively preserved OCT structure in the rod-predominant area. The presence of rod-specific retinal guanylase cyclase-2 is a possible explanation for the relative preservation of rod function in the *GUCY2D* mutation [34]. 

### 4.2. RPE65

The *RPE65* gene on chromosome 1p31.2 encodes retinoid isomerase, which is a 65 kDa enzyme expressed abundantly in RPE and is responsible for vitamin A metabolism in the retinoid cycle [35]. This deficiency results in a lack of 11-cis-retinal regeneration. The *RPE65* mutation accounts for 4–16% of LCA cases, which is relatively rare in the Chinese population but more prevalent in the Caucasian and Indian populations [36,37,38]. The common phenotype of *RPE65*-associated LCA includes profound nyctalopia at birth; however, some residual cone-mediated vision has been observed in these cases. Therefore, possibly an alternative supply of 11-cis-retinal, apart from *RPE65*, in cone photoreceptors has been proposed [15]. About 10% of *RPE65*-associated LCA cases present with vision worse than counting fingers initially, and transient improvement could be noted in early life. However, visual impairment gradually progresses from the second to the third decade of life [13,39,40]. Patients that carried at least one nonsense variant had more progressive deterioration in retinal sensitivity [41]. Myopia and cataract, mostly posterior subcapsular cataract (PSC), are ocular features related to the *RPE65* mutation [14]. Depigmentation and whitish deposits outside the macula, bull’s-eye maculopathy, or diffuse retinal degeneration are noted, and autofluorescence is typically reduced [14,42,43]. Pigmentary retinopathy can occur in the late stage [14]. Preserved ONL in the central island is observed on OCT, but ONL thickness is reduced adjacent to the fovea. The relatively preserved cone photoreceptors are explanatory to the cone-mediated vision [44,45]. Thinning of the retinal thickness, especially the ONL layer, and loss of lamination in the retina are more prominent in patients older than 30 years of age [14,44]. Hyperreflective deposits above the RPE may be found in correspondence to white dots in fundoscopy [7]. A diminished rod response is observed on the ERG, while a residual cone response has been recorded in some cases [40]. 

### 4.3. CRB1

The *CRB1* gene on chromosome 1q31.3 encodes Crumbs homologue 1, which is expressed in the inner segment of the photoreceptors and Muller cells to maintain adequate morphogenesis and polarity in retinal development [46]. Therefore, mutations in the *CRB1* gene have been associated with multiple retinal dystrophies, including RP, foveal retinoschisis, macular dystrophy, and LCA. Mutations within the Ca^2+^-binding EGF-like domain are often associated with more severe disease [10,47]. Approximately 9–17% of LCA cases have been related to *CRB1* mutations, which are higher in the Chinese population [15,23]. *CRB1*-associated LCA cases present relatively preserved visual function, nyctalopia, and high hyperopia [47]. The typical findings are progressive macular atrophy with nummular pigmentation and preserved para-arteriolar RPE [48,49]. Similar to the presentation of *CRB1*-associated RP cases, coats-like vasculopathy could also be present in LCA with *CRB1* mutations [50]. Thickening of the retina with loss of distinct lamination is a characteristic finding of *CRB1* mutation, which indicates retinal disorganization caused by developmental defects [48,49]. This unique OCT morphology differentiates *CRB1* mutations from other types of LCA and facilitates genetic diagnosis. The ERG response is generally diminished in these cases [49]. 

### 4.4. CEP290

The *CEP290* gene on chromosome 12q21.32 encodes a 290 kD centrosomal protein that localizes to the basal bodies of the photoreceptors connecting the cilium and is responsible for its formation, stability, and transportation function. Mutations in *CEP290* are associated with several systemic diseases, including Joubert syndrome, Senior-Løken syndrome, and Meckel syndrome [9]. It is also the most common cause of LCA in Caucasians, accounting for 15–30% of the cases, but relatively less prevalent in the Chinese population [23,51]. The most common variant, an intronic nonsense mutation, c.2991+1655A>G, was found in 77% of CEP-290-related LCA cases [51,52]. Vision is markedly impaired in *CEP290*-associated LCA cases in which the majority (62–89%) of patients had only light perception or less [13]. While nonsense mutations accounted for 75% of the variant of mutation in CEP290, patients with two nonsense mutations had a worse visual prognosis [53]. Photophobia and hyperopia are present. The fundus mostly exhibits a normal appearance in 74% of cases, but polymorphous white flecks, marbleized fundus, and pigmentary retinopathy may develop with age. A hyperautofluorescent ring can be found in the central macula [51,52]. Other associated fundus features include pseudopapillary edema, small atrophic RPE spot, chorioretinal atrophy, and Coats-like vasculopathy [54]. Apart from severe visual impairment at birth, the foveal architecture and integrity of the EZ are grossly preserved but are initially reduced in intensity on OCT images. ONL thickness in the paramacular region is decreased to one-third than the normal, and the inner retina is thickened and remodeled [13,14]. Cideciyan et al. reported that the foveal thickness decreased at a rate 0.7 to 1.3 µm per year in patients in their case series [13]. Cyst-like lesions were detected on OCT images in 43% of the cases reported by Pasalhika et al. [14]. Most of the cases with the *CEP290* mutation demonstrate an extinguished ERG response.

### 4.5. RDH12

Retinol dehydrogenase 12, which is encoded by the *RDH12* gene on chromosome 14q24.1, is expressed predominantly in the inner segment of the photoreceptor and participates in the retinoid cycle by converting 11-cis retinol to 11-cis retinal. Mutations in *RDH12* account for approximately 3–10% of LCA cases [9,15]. *RDH12*-associated LCA cases demonstrate poor but functional vision with transient improvement lasting less than 10 years. Progressive decline in vision occurs after the second decade of life [55]. Other related ocular features include nyctalopia and mild cataract, but not hyperopia. Fundus examination may reveal prominent atrophic maculopathy and peripheral pigmentation with profoundly reduced autofluorescence [56]. Garg et al. found that the peripapillary area was spared extensive macular atrophy in these cases [57]. A thinner retina in correspondence to macular atrophy and loss of laminar structure may be seen on OCT [58,59]. An extinguished ERG response under scotopic and photopic conditions has been consistently noted [56].

### 4.6. SPATA7

The *SPATA7* gene on chromosome 14q31.3 encodes a protein with a single transmembrane protein, termed spermatogenesis-associated protein 7. *SPATA7* is expressed in the cytoplasm of the inner segment of photoreceptors of mature retina in animal models, but its function in the visual cycle has not been fully elucidated [60]. The mutation in *SPATA7* has been identified not only in LCA but also in juvenile RP cases, while the severity of visual impairment largely differs [60]. The prevalence of *SPATA7* has been reported to be low as 3% in LCA cases [15]. Perrault et al. and Wang et al. reported two case series with nonsense mutations in *SPATA7*, which had a phenotype resembling *RPE65*-associated LCA. The cases presented with stationary poor vision, nystagmus, nyctalopia, and hyperopia. Fundus examination revealed peripheral chorioretinal atrophy, bone spicule pigmentation, and a central foveal hyperautofluorescent ring. ERG was extinguished in both the rod and cone responses [60,61].

### 4.7. AIPL1

The *AIPL1* gene on chromosome 17p13.2 encodes Aryl-hydrocarbon-interacting-protein-like 1, which is a cochaperone specific to photoreceptors that stabilize retinal cGMP phosphodiesterase in the phototransduction process. Both the rod and cone photoreceptors express the *AIPL1* gene during development, but only the rod photoreceptor continues its expression in the adult retina [9]. Missense and stop mutations (class I and II) in *AIPL1* produce an LCA phenotype, while a frameshift mutation (class III) results in the milder phenotype of juvenile RP [9]. Mutations in *AIPL1* account for about 5% of LCA cases. The variant p.W278X (c.834G>A) has been commonly reported [15,62,63]. The phenotype of *AIPL1*-associated LCA cases is considered to be severe, with rapidly progressive visual impairment to only light perception in most patients. Patients often present with nystagmus, photophobia, photoattraction, keratoconus, and PSC [64]. Although pigmentary retinopathy in the mid-periphery and central maculopathy may develop at a young age, evidence of reduced but residual autofluorescence in the posterior pole is reported at an early stage [65,66,67]. Tan et al. reported two 2-year-old patients without pigmentation in the fundus and preserved macular autofluorescence [66]. In addition to severely affected vision and macular appearance, OCT in younger patients shows a relatively preserved paracentral outer retina but loss of integrity of the EZ. The disorganization of the retinal structure progresses with age [14,65,68]. Extinguished ERG has been reported in most cases but could be partially preserved in a scotopic response at a young age as well [67]. Heegaard et al. reported a unique case of an extensive avascular retina at the periphery and torturous retinal vessels with a novel H82Y (244C>T) variant in the *AIPL1* mutation [69]. 

### 4.8. LCA5

The *LCA5* gene on chromosome 6q14.1 encodes lebercilin, which is expressed widely in the tissues composed of cilia, including photoreceptors. However, mutations in *LCA5* have been identified to be confined to retinal dystrophy without systemic association, while most of the cases meet the diagnosis of LCA [70,71]. The *LCA5* mutation accounts for only 1–2% of the LCA cases [15]. *LCA5*-associated LCA cases present with impaired vision, nyctalopia, and severe hyperopia. Fundus examination typically reveals diffuse mottling of the RPE at the periphery when aging and a frankly normal macula [72]. Jacobson et al. and Mackay et al. reported reduced autofluorescence in correspondence to RPE atrophy, but central hyperautofluorescence could be present [71,73]. Scattered RPE white dots, which could also be found in the *RPE65* phenotype, were common in the case series presented by Mackay et al. [71]. A relatively preserved foveal photoreceptor and an EZ zone with loss of lamination in the eccentric retina have been observed in the OCT images of younger patients [73]. An ERG response was mostly undetectable in the reported cases, but a retained residual cone response had been observed, compatible with the fovea-preserving features in OCT and fundus autofluorescence [71]. However, Mohamed et al. reported a dramatically different fundus feature in three of the five LCA cases in a Pakistani family confirmed with the *LCA5* mutation, which exhibited coloboma-like macular atrophy [74].

### 4.9. RPGRIP1

The protein product of the *RPGRIP1* gene on chromosome 14q11.2 regularly binds to the RP GTPase at the C-terminal interacting domain, to anchor on the connecting cilium in the retinal photoreceptor cells [9,75]. Mutations in *RPGRIP1* account for approximately 5% of LCA cases when null mutations occur, while less severe cone-rod dystrophy results from variants causing missense mutations [76]. *RPGRIP1*-associated LCA cases present with gradually declining impaired vision, photophobia, and nystagmus. Attenuated vessels, chorioretinal atrophy, and peripheral pigmentation may be observed on fundus examination in childhood [77]. Granular hyperautofluorescence corresponding to atrophic RPE is seen on fundus autofluorescence. [75,78]. OCT images usually demonstrate a thinner ONL and disorganized EZ, especially parafoveal [78,79]. However, Miyamichi et al. reported better retention of retinal structure on OCT before the age of 5 in their case series [78]. In previous reports, despite the relative foveal sparing on OCT and fundus examination, cone response was generally diminished on ERG, but rod function remained detectable in some younger cases [77,78]. 

### 4.10. CRX

The cone-rod homeobox (*CRX*) gene on chromosome 19q13.3 encodes a homeobox transcription factor, which is essential for early ocular development by regulating the differentiation and maintenance of photoreceptor cells [9,80]. The mutation in *CRX* accounts for up to 1% of LCA cases, which is autosomal dominant [9]. The typical phenotypes include stationary poor vision, night blindness, and frank macular disorganization in fundus examination, even in very early childhood [81,82]. OCT revealed a thinner thickness with a relatively preserved outer nuclear layer, and ERG was extinguished in both rod and cone responses in a case series [82]. Koenekoop et al. reported a case of gradual visual improvement sustained in the first decade of life with a measurable cone response on ERG, which was undetectable in infancy [83]. 

### 4.11. NMNAT1

The *NMNAT1* gene on chromosome 1q36.22 encodes a nuclear isoform of NAD+-synthesizing nicotinamide mononucleotide adenylyltransferase. Besides affecting NAD+ synthesis for energy supply in the retina, deficiency in *NMNAT1* is also evident in poor retinal differentiation and impaired neuroprotective function [84]. Mutations in *NMNAT1* account for 2.3–4.9% of LCA cases [85,86]. The *NMNAT1*-associated LCA cases have severely impaired vision at birth and coloboma-like macular dystrophy since early infancy. Attenuated vessels, pallor optic disc, and peripheral pigmentation are observed on fundus examination. Extreme early onset and rapid progression are characteristics of *NMNAT1* mutations [84,87]. Damage from light exposure possibly explains the predominant central retinal degeneration. Retinal disorganization in all layers with a well-defined boundary between normal and abnormal retinas has been found in the available OCT study [84,87]. Extinguished ERG is typical for *NMNAT1* mutations. 

### 4.12. IMPDH1

The protein product of the *IMPDH1* gene on chromosome 7q32.1 is involved in the rate-limiting step of guanine synthesis. In addition, research on the IMPDH protein has revealed its binding capability to single-stranded nucleic acids, which possibly results in a retinal pathology through this pathway. *IMPDH1* is expressed in a variety of tissues but is highly expressed in the retinal tissue [9]. Mutations in *IMPDH1* were initially found in autosomal-dominant RP cases, while a more severe phenotype as LCA with an autosomal-dominant inheritance pattern has also been reported [88]. *IMPDH1*-associated LCA appears to be rare. However, the case series by Bowne et al. reported a prevalence of 8.3% (2/24) [88]. They reported two *IMPDH1*-associated LCA cases that presented with mottling or depigmented fundus. One of the patients also had systemic hypotonia and growth retardation [88]. 

### 4.13. RD3

The *RD3* gene on chromosome 1q32.3 is only expressed in retinal photoreceptors and is essential for the transportation of guanylate cyclase between the inner and outer segments [89]. Less than 1% of LCA cases have mutations in the *RD3* gene [15,90]. *RD3*-associated LCA cases present nyctalopia and poor vision at birth, with progressive decline to barely precepting light. The visual function is comparably reduced as in the *GUCY2D* mutation because of the sharing dysfunction in guanylate cyclase [91]. Central maculopathy is observed in early life with or without a bull’s-eye pattern on fundus examination, and pigmentary retinopathy with a hammer-beaten appearance develops in the third decade of life. A disorganized retina with reduced thickness in every layer is observed on OCT images. Extinguished ERGs are universally noted in these cases [92]. 

### 4.14. LRAT

Lecithin retinol acyltransferase is the product of the *LRAT* gene on chromosome 4q32.1, which converts all-trans retinol to all-trans retinyl esters in the RPE phase of the retinoid cycle. Mutations in the *LRAT* gene have been reported in approximately 1–2% of LCA cases, and their phenotype is similar to that of the *RPE65* mutation [15,93]. *LRAT*-associated LCA cases usually present with nyctalopia with poor vision but absence of photophobia. On fundus examination, a grossly normal but mild mottling appearance is observed in the posterior pole, and a demarcation margin with diffuse granular depigmentation can be seen outside the macula [93,94]. Borman et al. reported a case series using OCT, which revealed a thinner retinal thickness and loss of normal lamination in aged patients, but a preserved foveal architecture was found in a 27-year-old patient [93]. The rod response in ERG is always diminished, but a residual cone response has been detected in some cases [94]. 

### 4.15. TULP1

The tubby-like protein 1 is encoded by the *TULP1* gene on chromosome 6q21.31, which is expressed specifically in the retina and is localized in the cilia of the photoreceptor’s inner segment, including both rods and cones. Mutations in *TULP1* account for approximately 1% of LCA cases [9]. TULP-associated LCA cases present with poor vision, nystagmus, and nyctalopia since infancy, but no photophobia or hyperopia. Funduscopy findings remain unremarkable in early childhood, but pigmentary retinopathy is noted after the second decade of life, while maculopathy develops in the third decade [95]. A hyperautofluorescent ring can be seen in the central macula. OCT reveals a thinner retina with loss of outer retinal lamination eccentric to the fovea, while relatively preserved foveal bulge [96]. The ERG response is extinguished in most patients [95,96]. 

### 4.16. KCNJ13

The *KCNJ13* gene on chromosome 2q37.1 encodes protein Kir7.1, which is localized on the apical process of RPE and functions as a potassium channel [97]. LCA cases of mutations in *KCNJ13* present with nyctalopia, nystagmus, and cataracts [98]. Funduscopic findings are normal in early life, but characteristic dense pigmentary maculopathy develops after the first decade of life. A corresponding hyperreflective deposit spread in the disorganized retina can be seen on OCT. The ERG response is extinguished in most cases [98,99]. 

### 4.17. PRPH2

The *PRPH2* gene on chromosome 6p21.1 encodes peripherin 2, which is present in the disc rims of the outer segment of the photoreceptors. Mutations in *PRPH2* are related to RP, retinitis punctata albescens, and several macular dystrophies [100]. Mutation of *PRPH2* has been reported in LCA in a limited manner. Poor vision, nyctalopia, and nystagmus have been observed in the reported case [101,102]. Fundus examination was nearly normal in infancy, but pigmentary retinopathy and bull’s-eye maculopathy were present in adulthood [101]. The family members who were carriers also exhibited maculopathy, even though they were asymptomatic [102]. OCT revealed a relatively normal thickness but disrupted outer retina with hyperreflective deposits [101]. The ERG response was extinguished in the reported cases. 

### 4.18. IQCB1

The *IQCB1* gene on 3q13.33 encodes nephrocystin, which is localized in the cilia in the kidney and retinal photoreceptors and interacts with *CEP290* in ciliary function [103]. Patients with mutations in *IQCB1* have onset of LCA in early childhood, and some of them develop renal dysfunction in adolescence [104]. These patients have progressively impaired vision, nyctalopia, and high hyperopia. Fundus examination may reveal early hypo- and hyperpigmentation outside the vascular arcades in a lobular fashion, which is distinct from other LCA phenotypes [105]. Eccentrically decreasing persevered foveal ONL and loss of EZ integrity may be seen on OCT. An extinguished ERG response has been seen in reported cases [104]. 

Many LCA-associated mutations have a relatively lower prevalence, and limited cases have been reported in the literature. Mutations in *OTX2* and *GDF6* are more known for severe ocular malformations, such as microphthalmia, because of their essential role in embryonic ocular development; however, milder cases with retinopathy have been diagnosed as LCA as well [106,107]. Mutations in the *IFT140* gene cause ciliopathy, which is associated with retinal, renal, and skeletal abnormalities (Mainzer-Saldino syndrome) [108]. However, a specific genotype-phenotype correlation has not been established in the infrequent mutations. 

## 5. Therapeutic Approach

Gene augmentation therapy is the main source of potential treatment for LCA. Animal studies have been conducted on commonly involved genes, including *GUCY2D*, *RPE65*, *AIPL1*, *RPGRIP1*, *LCA5*, *CEP290*, and *RDH12*, mostly by using adeno-associated virus (AAV) vector-mediated or lentiviral-vector-mediated gene augmentation therapy [15]. Moreover, clinical trials focusing on *GUCY2D*, *RPE65*, and *CEP290* are ongoing [109]. To date, the most advanced progress has been made in the treatment of *RPE65* mutations [110,111,112,113]. Among them, Luxturna™ (Voretigene neparvovecryzl; Spark Therapeutics, Philadelphia, PA) is the first treatment to undergo a phase III trial and is approved by the U.S. Food and Drug Administration in genetic therapy for LCA [112]. Luxturna™ is an AAV designed to be administered to the sub-retinal space through the pars-plana approach, which carries the *RPE65* gene to restore the deficiency in the *RPE65* protein in patients with LCA with a biallelic *RPE65* mutation. Early intervention is essential for success because more retinal cells may be viable during early life [113]. Improvements in visual acuity, perimetry, and retinal sensitivity have been reported and were sustained until 4 years after treatment in the latest clinical trials [114]; however, ongoing retinal degeneration was still noted with extended follow-up [110,111]. 

Being one of the most identified variants in western countries, gene therapy trials have also focused on c.2991+1655A>G in the *CEP290* gene. A pioneer CRISPR (clustered, regularly interspaced, short palindromic repeats)-mediated gene editing therapy focusing on the c.2991+1655A>G variant has been launched for phase I clinical trials. Another approach with intravitreal injection of mRNA, which serves as an anti-sense oligonucleotide for genetic editing, has been introduced to restore the expression level of *CEP290* (NCT03140969) [115,116]. Pharmacotherapy could improve the retinoid cycle dysfunction by providing a specific substrate. A clinical trial of QLT091001, a precursor of 9-cis retinol taken orally, showed improvement in vision and perimetry in *LRAT*- and *RPE65*-associated LCA cases [117]. Stem-cell therapy is considered a rescue therapy for cases with atrophic macula by introducing progenitor retinal cells. It has been proved that both embryonic stem cells and induced pluripotent stem cells are capable of regenerating retinal tissue for potential treatment of RP and age-related macular degeneration. Cell replacement therapy for atrophic retina is ubiquitous in different causative retinal diseases and is promising in the treatment of LCA and various inherited retinal diseases [118]. 

## 6. Conclusions

LCA is the most severe and early onset form of IRDs and is a major cause of childhood blindness. Hence, it is important to find an effective treatment soon. However, its highly heterogeneous phenotypic and genetic spectra have made it difficult to diagnose and plan individualized treatment. Owing to the modern advances in next-generation sequencing, the efficiency and accuracy of identifying causative gene mutations have been largely elevated. Therefore, the genotype-phenotype correlations can be explored more precisely when incorporating clinical assessments and image examinations. Nonetheless, the correlations between phenotypical features and intra-genetic variability or ethnical difference are even less established and further research is required in the future. Here, we provide a comprehensive review, anticipating to enhance the diagnosis and treatment of potential LCA cases in clinical practice, as well as the development of available therapeutic interventions. 

## Figures and Tables

**Table 1:** Overview of the genes responsible for LCA and their clinical presentation, including the common funduscopic features, exception in ERG, and findings in OCT *.

Locus Name		Causative Gene	Chromosome Site	Encoded Protein	Protein Function	Frequency	Typical Fundus	ERG **	OCT Features
LCA 1		*GUCY2D*	17p13.1	Retinal guanylate cyclase-1	Photo-transduction	6~21%	Normal appearance until central RPE change in third decade	Rod- preserved	Preserved EZ and ONL with reduced signal intensity
LCA 2		*RPE65*	1p31.2	Retinoid isomerase	retinoid cycle	4~16%	Normal macula with white dot and depigmentation in mid-periphery	Cone- preserved	Preserved EZ and ONL but degenerated in 3rd decade
LCA 3		*SPATA7*	14q31.3	Spermatogenesis-associated protein 7	Ciliary transportation	3%	Pigmentary retinopathy in mid-periphery, chorioretinal atrophy		
LCA 4		*AIPL1*	17p13.2	Aryl-hydrocarbon-interacting-protein-like 1	Photo-transduction	5%	Central atrophic maculopathy with peripheral pigmentary retinopathy	Rod- preserved	Loss of EZ and outer retinal lamination
LCA 5		*LCA5*	6q14.1	Lebercilin	Ciliary transportation	1~2%	Normal macula with white dot and mottling RPE in mid-periphery	Cone- preserved	Preserved in fovea but loss of outer retinal lamination eccentrically
LCA 6		*RPGRIP1*	14q11.2	RP GTPase regulator-interacting protein 1	Ciliary transportation	5%	Pigmentary retinopathy in mid-periphery	Rod- preserved	Loss of EZ and outer retinal lamination eccentrically
LCA 7		*CRX*	19q13.3	Cone-rod homeobox	Photoreceptor morphogenesis	1%	Central atrophic maculopathy		Thinner retina with preserved ONL
LCA 8		*CRB1*	1q31.3	Crumbs homologue 1	Photoreceptor morphogenesis	9~17%	Central atrophic maculopathy with dense pigmentation, preserved para-arteriolar RPE, Coats-like vasculopathy		Thicken and disorganized retina in macula
LCA 9		*NMNAT1*	1q36.22	Nicotinamide nucleotide adenyltransferase 1	NAD biosynthesis	<5%	Central atrophic maculopathy with peripheral pigmentary retinopathy		Disorganized retina in macula
LCA 10		*CEP290*	12q21.32	290 kDa centrosomal protein	Ciliary transportation	15~30%	Normal macula, white dot and mottling RPE in mid-periphery, Coats-like vasculopathy		Preserved EZ in fovea, remodeled inner retina and loss thickness eccentrically
LCA 11		*IMPDH1*	7q32.1	Inosine 5’-monophosphate dehydrogenase 1	Guanine synthesis	8%	Mottling and depigmentation in macula and periphery		
LCA 12		*RD3*	1q32.3	Retinal degeneration 3	Photo-transduction	<1%	Central atrophic maculopathy with peripheral pigmentary retinopathy		Disorganized retina in macula
LCA 13		*RDH12*	14q24.1	Retinol dehydrogenase 12	retinoid cycle	3~10%	Central atrophic maculopathy with peripheral pigmentary retinopathy		Disorganized retina in macula
LCA 14		*LRAT*	4q32.1	Lecithin:retinol acyl transferase	retinoid cycle	1~2%	Nearly normal but mottling macula with granularity in mid-periphery	Cone- preserved	Loss of EZ and outer retinal lamination in older cases
LCA 15		*TULP1*	6q21.31	Tubby-like protein 1	Ciliary transportation	1%	Normal in childhood but central atrophic maculopathy and pigmentary retinopathy in mid-periphery in third decade		Preserved fovea but loss of outer retinal lamination eccentrically
LCA 16		*KCNJ13*	2q37.1	inwardly rectifying potassium channel (Kir) 7.1	Photo-transduction	Unknown	Central atrophic maculopathy with dense pigmentation		Disorganized retina in macula with pigment clump
LCA 18		*PRPH2*	6p21.1	Peripherin 2	Photoreceptor morphogenesis	Unknown	Maculopathy and pigmentary retinopathy in mid-periphery		Loss of EZ and outer retinal lamination
		*IQCB1*	3q13.33	IQ motif-containing B1 protein	Ciliary transportation	Unknown	Lobular hypo-hyper pigmentation in mid-periphery		
	Other causative genes for LCA ***			*ALMS2*, *CABP4*, *CCT2*, *CEP164*, *CLUAP1*, *CNGA3*, *DTHD1*, *GDF6* (LCA 17), *IDH3A*, *IFT52*, *IFT140*, *MCOR*, *MERTK*, *MYO7A*, *NPHP3*, *NPHP4*, *OTX2*, *TUBB4B*, *USP45* (LCA 19), *ZNF423*					
	Syndromic type of LCA			Senior-Løken syndrome (*CEP290*, *IQCB1*), Joubert syndrome (*CEP290*), Meckel syndrome (*CEP290*), Klippel-Feil syndrome (*GDF6*), Usher syndrome (*MYOA7*), syndromic microphthalmia (*GDF6*, *OTX2*)					

Abbreviations: LCA, Leber’s congenital amaurosis; ERG, electroretinogram; OCT, optical coherent tomography; EZ, ellipsoid zone; ONL, outer nuclear layer. * Inheritance patterns in the genes listed in the table are autosomal recessive except for *CRX*, *IMPDH1*, and *OTX2*. ** Exceptions from the typically diminished ERG. *** Other genes found in case reports to be responsible for LCA on the OMIM website [17], but without a conclusion on the phenotypic features.
